# Comparative assessing prefabricated and conventional 3D morphology carbon emission intensity of economy chain hotel buildings in Hangzhou

**DOI:** 10.1038/s41598-026-55637-z

**Published:** 2026-06-11

**Authors:** Dongyu Liu, Pengcheng Du, Qian Wu, Lena Tran, Yin Lou, Wenwen Shi, Gangwei Cai, Weijun Gao

**Affiliations:** 1 School of Civil Engineering and Architecture, Zhejiang University of Science and Technology, Hangzhou, China; 2 Jinshi Engineering Design Shandong Group Co., Ltd., Shandong, China; 3 Shaoxing Keqiao District Science and Technology Innovation Service Center, Zhejiang, China; 4 School of Culture Communication & Design, Zhejiang University of Finance and Economics Dongfang College, Hangzhou, China; 5 Faculty of Built Environment and Surveying, Universiti Teknologi Malaysia, Skudai, Malaysia; 6 Institute of Landscape Architecture, Zhejiang University, Hangzhou, China; 7 Department of Architecture, The University of Tokyo, Tokyo, Japan; 8 Zhejiang Urban Governance Studies Center, Hangzhou, China; 9 College of Artificial Intelligence, Zhejiang University, Hangzhou, China; 10 Faculty of Environmental Engineering, University of Kitakyushu, Fukuoka, Japan; 11 The Engineering Academy of Japan, Tokyo, Japan

**Keywords:** Prefabricated construction, Carbon emission intensity (CEI), Economy chain hotel, Building 3D Morphological, Life cycle assessment (LCA), Engineering, Environmental sciences

## Abstract

Understanding the influence of building morphology on carbon emissions is essential for developing effective low-carbon strategies in the hospitality sector. This study investigates the carbon emission intensity (CEI) of 200 economy chain hotels in Hangzhou, China, constructed using comparative both prefabricated and conventional methods, from a three-dimensional morphological perspective. A comprehensive life cycle assessment (LCA) framework was adopted, encompassing material production, transportation, construction, renovation, and demolition stages. The analysis explores the relationship between 3D morphological characteristics and CEI under the two construction systems. Results indicate that prefabricated hotels generally exhibit greater carbon reduction efficiency, particularly when applied to regular geometric configurations such as rectangular and polygonal layouts. Additionally, a consistent positive correlation was found between building shape factor and CEI in both prefabricated and traditional constructions, highlighting the critical role of compact architectural form in minimizing lifecycle carbon emissions.

## Introduction

In recent years, the challenges of greenhouse gas emissions and climate change have become central concerns within the framework of global sustainable development^1–3^. As one of the world’s largest emitters, China’s carbon reduction strategies have drawn significant international attention. In September 2020, the Chinese government set forth the dual targets of peaking carbon dioxide emissions by 2030 and attaining carbon neutrality by 2060. Subsequently, in September 2021, the State Council released a series of guiding policy documents to support these commitments, offering concrete directions for realizing both carbon peaking and neutrality^4,5^. In recent years, China’s rapid urbanization has fueled the continuous expansion of the construction sector. However, this accelerated growth has also led to intensive consumption of materials and energy, as well as considerable carbon emissions throughout the life cycle of hotel buildings, including construction, operation, and demolition. To address these environmental challenges, China has actively promoted prefabricated construction methods and technologies. According to the 14th Five-Year Plan for Building Industry Development released by the Ministry of Housing and Urban-Rural Development, prefabricated buildings are expected to represent more than 30% of all new construction projects by 2025.

**Fig. 1:**
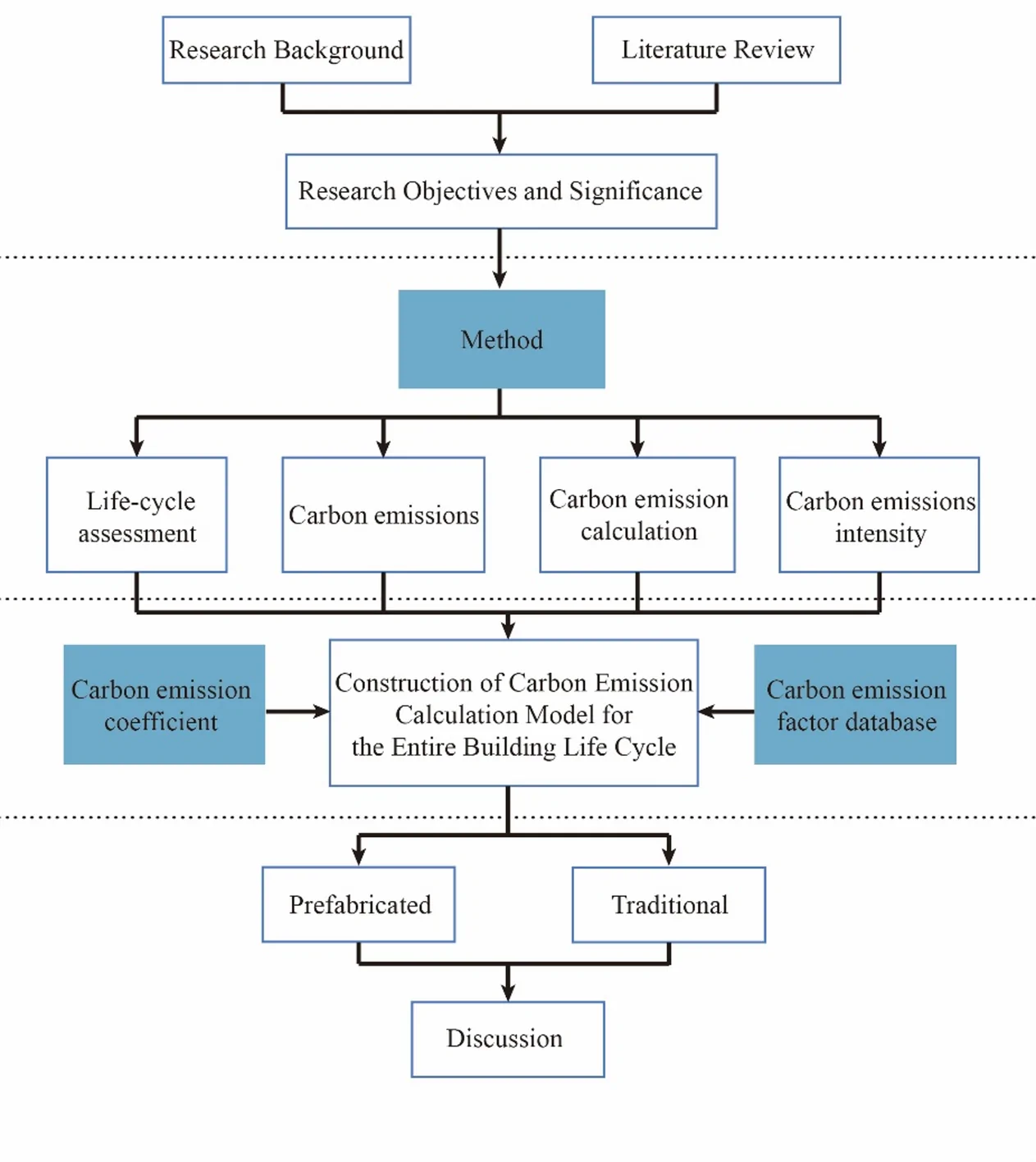
The technical route framework for research.

In 2023, Ctrip revealed data at Global Partner Conference: the global average daily carbon emissions in the first half of 2023 were 27 million tons, with 11% coming from the tourism industry, and the hotel industry’s carbon emissions accounted for 60% of the total emissions from the tourism^6^.《2024 China Urban-Rural Development Carbon Emissions Research Report (2024 Edition)》^7^ reveals that in 2022, the total construction-related carbon emissions from buildings and the construction industry nationwide reached 5.13 billion tonnes of CO₂ (tCO₂), accounting for 48.3% of China’s energy-related carbon emissions. In China, hotel buildings account for a considerable proportion of the industry. By the end of 2022, the number of accommodation facilities in China has reached 430,000, hotel facilities account for 279,000, with a total of 14.264 million rooms, the proportion of economy hotels (two star and below) is 78.51%^8^. Against this backdrop, it is imperative to conduct carbon emission measurement and research on prefabricated hotel buildings. This study aims to systematically analyze the carbon footprint across the entire lifecycle of such structures, providing data-driven insights to support low-carbon transformation in the construction sector. This study analyzes data from 200 economy chain hotels in Hangzhou to establish a hotel building morphology database and quantify the carbon emission differences between conventional hotels (pre-renovation) and prefabricated hotels (post-renovation). The relationship between carbon emissions and three-dimensional building morphology was examined through computational modeling, which revealed correlations between morphological indicators and carbon emission intensity. Compared to previous hotel carbon emission studies, which have predominantly focused on the operational stage or single construction methods, the novelty of this research lies in that it treats the three-dimensional geometric form of buildings (shape coefficient) as a core explanatory variable and quantitatively analyzes its association with CEI, moving beyond the conventional focus on materials or technologies alone. In addition, it systematically compared the carbon emissions of prefabricated and traditional construction methods for different building forms within the same group of hotel samples, emphasizing the importance of construction methods. Results demonstrate that prefabricated construction offers significant advantages in hotel form renovation, particularly for buildings with complex geometries, where it outperforms conventional construction in achieving low-carbon outcomes. These findings provide valuable guidance for promoting prefabricated, low-carbon renovations in economy chain hotels and contribute to advancing the industry’s overall low-carbon transition (Fig. 1).

## Literature review

### Life cycle stage classification

Life cycle assessment (LCA) is a key method for evaluating the environmental impact of a product, process, or service throughout its entire lifecycle. Concepts related to life-cycle carbon emission assessment (LCCEA) include life-cycle assessment and life-cycle energy assessment (LCEA)^9^. Currently, most researchers use the LCA to calculate building carbon emissions. LCA originated in the 1970s and is a method for assessing and quantifying the environmental impact of a product’s production, use, and ultimate disposal, which is considered a “cradle-to-grave” process^10^. The standard is derived from ISO 21,930, issued by the International Organization for Standardization (ISO) in 2017. According to ISO 21,930, the entire life cycle of a building is divided into five stages and 17 sub-stages: material production (A1–A3), construction (A4–A5), use (B1–B7), end-of-life (C1–C4), and additional information beyond the system boundary (D)^11^. In other papers, different scholars also gave different stage divisions. On the other hand, SDGs including low-carbon, resilient, sustainable tourism and others^12–16^.

Leif Gustavsson^17^ divides the life cycle of wooden structure hotels into four stages for carbon emission assessment: material production, on-site construction, operation, and dismantling. Huanyu Wu^18^ emphasizes building decoration and evaluates emissions across materialization, transportation, decoration, operation, and dismantling stages, highlighting the dynamic characteristics of decoration-related carbon emissions. Usha Iyer-Raniga^19^ and Zujian Huang^20^ classify the building life cycle into four stages: material production, construction, use and maintenance, and material landfilling at the end-of-life phase Wahidul K. Biswas^21^ and C.K. Chau^9^ summarize it into five stages: planning, construction, operation, renovation, and disposal. Although the division of stages varies across studies, the core consistently encompasses the entire process from building material production to building disposal. This study aims to highlight the embodied carbon emissions in economy chain hotel buildings that require frequent renovation and refurbishment; therefore, it adopts a five-stage framework system boundary consisting of production, transportation, construction, renovation, and demolition. Its definition, coverage of activities, and corresponding relationship with ISO 21930:2017 standard are shown in Table 1:

**Table 1:** Stage division and its corresponding relationship.

Division of research stages	Major activities	ISO 21930
Production	The production of main building materials	A1-A3
Transportation	The transportation of materials from the production site to the construction site	A4
Construction	Energy consumption of machinery and manual activities	A5
Renovation	Secondary decoration	B2
Demolition	Demolition of buildings and disposal of waste	C1-C4

Several scholars have applied LCA to compare carbon emissions between prefabricated and conventional construction like modular and AI^22–25^. Aisan Kong^26^ examined emissions during the construction of prefabricated floors and compared the results with conventional methods, finding that prefabrication can reduce emissions by 35% under fixed transportation distances and emission factors. Fang Zhou^27^ conducted a comparative analysis and concluded that prefabricated buildings reduce carbon emissions by approximately 86 kg per square meter relative to conventional construction. Omar^28^ employed LCA to analyze a typical two-story residential building in Malaysia, contrasting conventional and prefabricated approaches. Hamza Pervez^29^ compared prefabricated and concrete buildings, reporting that prefabricated construction lowers greenhouse gas emissions by 46.9% compared to conventional concrete structures. Pons^30^ assessed three prefabricated building types against a conventional concrete building and showed that a prefabricated steel structure reduces life cycle emissions by 1,021,359 kgCO₂eq/m². Collectively, these studies demonstrate that prefabricated buildings consistently exhibit carbon reduction advantages over conventional buildings, particularly in materialization, renovation, and dismantling stages.

The role of LCA in this research provides a rigorous methodological structure that integrates carbon emission calculation across all non-operational stages. By focusing on the renovation stage—often overlooked in LCA studies—this framework specifically addresses the frequent refurbishment cycles characteristic of economy chain hotels, thereby capturing the often-underestimated embodied carbon emissions that are central to the hotel industry’s low-carbon transition.

### Hotel carbon emissions calculation

Substantial advances have been achieved in the sustainable development research of the tourism and hospitality sector, particularly regarding the decarbonization of hotel buildings^31–34^. In the study of carbon emissions for hotel buildings, Viachaslau Filimonau^35^ measured the carbon emissions of two hotel buildings in the UK through the LCA, proving the feasibility of using the LCA in hotel buildings. Scheuer^36^ used LCA to evaluate the energy consumption and greenhouse gas emissions of a university building with hotel functions in the US. Wu^37^ determined the carbon emission level of hotels by collecting data of hotels. Ebunu^38^ calculated the carbon emissions of five hotels in Sri Lanka by multiplying the collected data on the consumption of diesel, water, electricity and other resources of the hotels by the corresponding carbon emission factors. Jing Qiu^39^ used the LCA to measure the carbon emission reductions of different star-rated hotels in different provinces in China during the green renovation process. The results show that the operation stage is the stage with the highest carbon emissions in the hotel building life cycle. For prefabricated hotels, Cristina Campos^40^ evaluated the environmental impact of three prefabricated hotels in coastal and mountainous areas of Spain and Portugal by LCA. Kuo^41^ studied the environmental impact of prefabricated hotels in Taiwan.

Contemporary studies increasingly highlight the interconnection between spatial/ mental design, construction technology, and environmental resilience in achieving long-term sustainability goals^2,42–46^. From the perspective of the life cycle of hotel buildings, carbon emissions in the operation phase account for approximately 60-87.7% of the total carbon emissions^47–50^. Joseph H.K. Lai^51^ collected data from a typical hotel in Hong Kong and used the carbon emission factor method (multiplying the energy consumption by the corresponding carbon emission factor) to measure the carbon emissions of the hotel during operational period. Ultimately, it was measured that the daily carbon dioxide emissions per room are 31.07 kg. Some scholars^52,53^ showed that there is a significant difference between the total carbon emissions in the hotel life cycle and the carbon emissions in the operation phase.

In summary, existing research on carbon emissions from hotel buildings has predominantly focused on energy consumption and carbon emissions during the operational phase^54^. Discussions regarding the non-operational phases, however, often treat them as a whole or rely on rough estimates, lacking a detailed breakdown and in-depth analysis of sub-stages such as material production, transportation, construction, decoration, and demolition^55,56^. Nevertheless, for the specific typology of economy chain hotels, their business model is characterized by frequent renovations and short refurbishment cycles. This implies that over the building’s whole life cycle, the cumulative contribution of embodied carbon emissions—central to the non-operational phase and driven by activities such as decoration and renovation—may be significantly underestimated.

Therefore, this study shifts perspective by systematically focusing on the carbon emissions during the non-operational life-cycle stages of hotel buildings. It aims to conduct a detailed accounting of the carbon footprint across stages including material production, transportation, construction, decoration, and demolition, thereby revealing the distribution and scale of emissions at each phase. This approach not only seeks to address gaps in the existing literature but also responds to the practical need in economy hotel management for a scientific assessment of the carbon costs associated with “renovation and refurbishment.” Ultimately, it provides a more comprehensive data foundation and strategic basis for the low-carbon operation, maintenance, and renewal of hotel assets.

### CEI and building morphology

From the perspective of individual buildings, existing research does not have a unified standard for calculating CEI^57–59^. Some researchers calculate the CEI by computing the energy efficiency during the building’s life cycle^29,60–62^. Other scholars have also used other methods to calculate the CEI of buildings. Since the 1990s, the international definition of the energy usage index (energy intensity) for the hotel industry has been the energy consumption of per unit of building area(kgCO₂/m²)^63,64^. D.Z. Li^65^ calculated the life cycle carbon emissions of a residential building in Nanjing, and constructed a CEI model. Rodrigo Schons Arenhart^66^ mentioned that the CEI of hotels is the ratio of carbon emissions to the number of rooms༈kgCO₂/rooms༉. Others studied the methods for calculating carbon intensity and pointed out that normalizing carbon emissions to the building area using the unit area carbon emission method can effectively compare the carbon intensity between different buildings^67–70^. In summary, the application and calculation methods of the CEI are relatively extensive, which is conducive to optimizing decision-making. Sustainable development within the tourism and hospitality sector has increasingly been aligned with the United Nations Sustainable Development Goals (SDGs), particularly SDG 11 on sustainable cities^71,72^.

It is crucial to discuss design 3D building morphology (such as shape coefficient and extreme environment habitats)^73–77^. The morphology of buildings strongly impacts CEI^78^. Weijia Feng^79^ have pointed out that the larger the average aspect ratio of a building and the higher the average number of floors, the greater the total energy consumption of the building. Yang Wang^80^ employed a spatial regression model to analyze the quantitative impact of urban 3D building morphology on per capita electricity consumption. Suzi Dilara Mangan^81^ analyzed data such as the typology, plan type, and number of floors of buildings in cities to explore their impact on building energy efficiency. In terms of reducing carbon emissions for buildings, current research mainly focuses on the material transformation of buildings, with less consideration of factors related to building morphology^82–84^, or new methods like 3D printing^85^. For example, Gan utilized simulations to optimize the layout of a 40-story housing project in Hong Kong, achieving a reduction in total energy usage of up to 30–40%^86^. Therefore, current research seldom starts from the perspective of building morphology to achieve the goal of carbon emission reduction. This study aims to reveal the association between three-dimensional building morphology and carbon emissions. Adopting building volume as the functional unit allows the dependent variable to be directly linked, in terms of mathematical dimensionality, with the key independent variable (S/V), thereby enabling a more straightforward exploration of the impact mechanism of architectural form on carbon emission efficiency. Furthermore, the system boundary of this research excludes carbon emissions from the operational stage, focusing instead on emissions attributable to the building’s physical entity (structure, envelope, and interior finishes). These emissions fundamentally originate from the material substances that constitute the built space. Consequently, this study ultimately selects carbon emission intensity per unit volume (kgCO₂/m³) as the core metric. This normalized metric allows fair comparison of environmental performance across hotels of different sizes and floor areas (Table 2).

**Table 2:** Comparison of different standardization methods for building carbon emission intensity.

Method	Applicable scene	Association
Unit Area (kgCO₂/m²)	Operating energy consumption	Widely used but confined to two-dimensional analysis, it is disconnected from three-dimensional morphological studies and susceptible to interference from variations in floor height.
Unit Room (kgCO₂/Room)	Carbon footprint of hotel services	It completely detaches from the physical form of the building and is more suitable for the operational stage.
Unit Value (kgCO₂/yuan)	Macro analysis of the industry	Governed by numerous non-physical factors, it cannot be used for design and technological comparisons.
Unit Volume (kgCO₂/m³)	Carbon emission, morphological comparison	It aligns with three-dimensional geometric metrics, directly reflects the material metabolism of the physical entity, and provides a pure physical benchmark for comparison.

## Method

### Data acquisition

The research focuses on 200 economy chain hotels in Hangzhou. Data were obtained from online hotel reservation platforms such as Ctrip, Dianping, and Qunar. At present, common approaches to data acquisition include Python-based rule programming and web crawler tools. After evaluation, web crawler tools were selected for their broader functionality and ease of use. Specifically, this study employed Octoparse, a web scraping software that extracts data from websites and converts them into structured formats such as Excel files or databases. Octoparse also provides cloud-based solutions that enable large-scale, precise, and efficient data collection. Its features include stable performance, high-speed multi-threading, customizable fields, and automated recognition of web page content, which facilitate rapid and user-friendly data extraction.

**Fig. 2:**
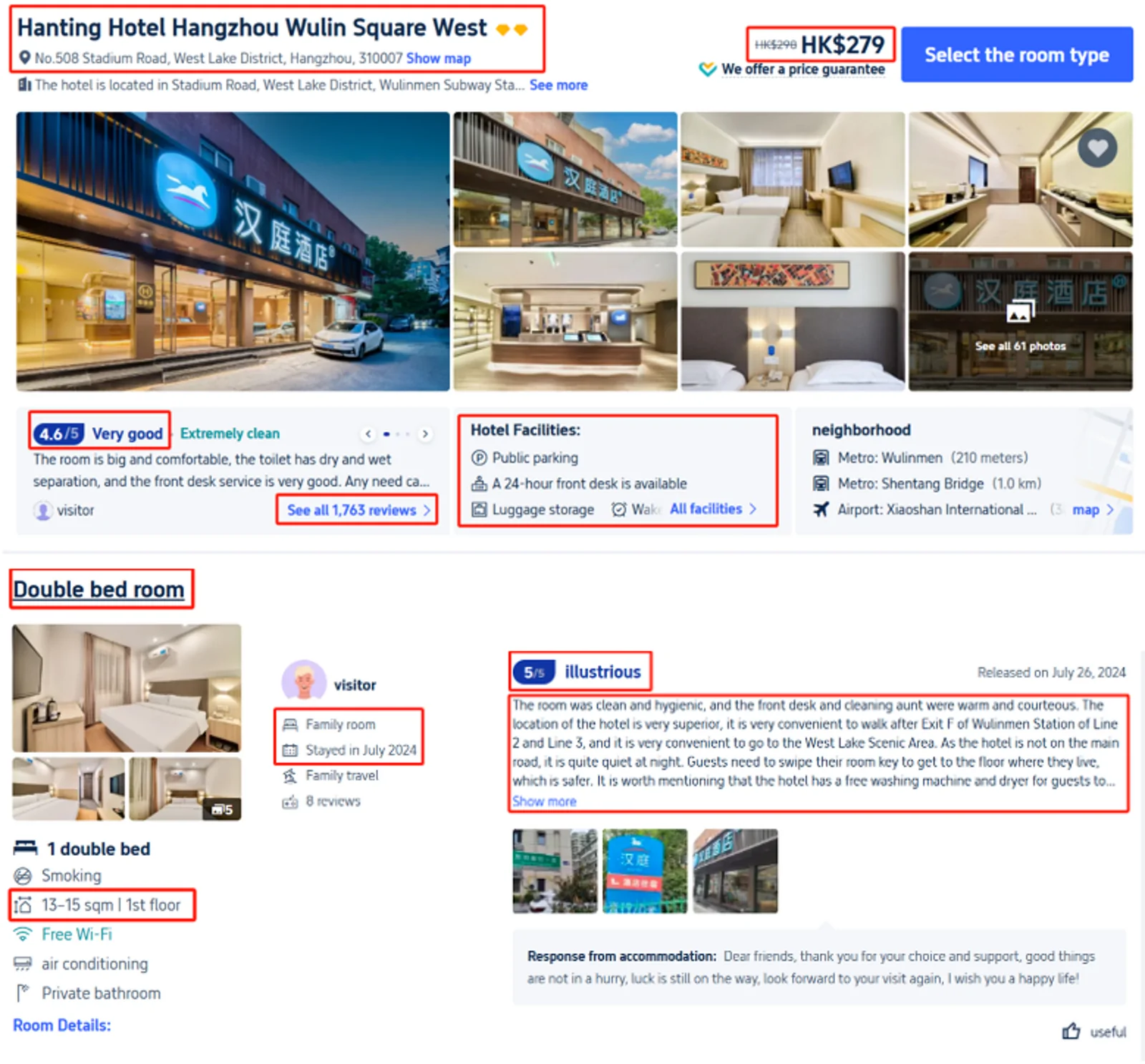
Example of web data crawling.

As shown in the Fig. 2, using the Octopus to scrape the hotel reservation page on Ctrip for hotel names, hotel locations, number of rooms, the lowest hotel booking prices, interior and exterior images of the hotel, the hotel’s comprehensive rating, the types of rooms and their corresponding areas, user check-in times, user comments, and user ratings for analysis. In addition, Baidu Maps can also be used to obtain street view images, satellite images, etc. of the hotel. Extract the hotel building form through satellite images, and then summarize the information in Excel.

To ensure data reliability, we implemented a rigorous data cleaning process. Information on room counts, pricing, and images for the same hotels was cross-verified across multiple platforms, including Ctrip, Meituan, and Baidu Maps. Data entries with obvious contradictions and severe outliers were systematically excluded. Subsequently, we selected a subset of hotel samples for further validation by cross-referencing building information with on-site inspections, thereby ensuring data accuracy and maintaining an acceptable margin of error.

### Hotel carbon emissions calculation method

#### Case study description

**Fig. 3:**
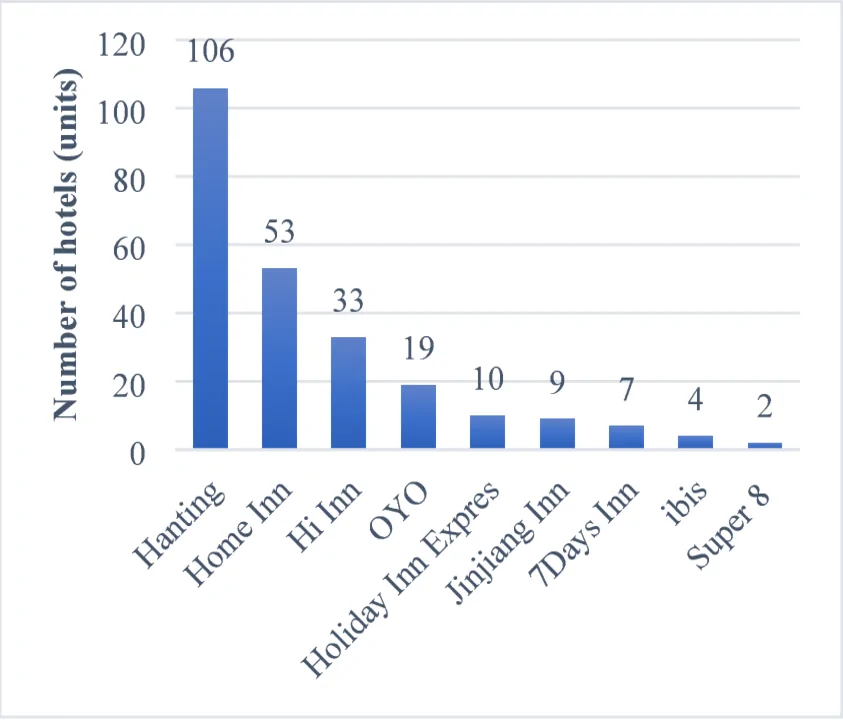
Number of economy chain hotels in Hangzhou (data as of March 2025).

**Fig. 4:**
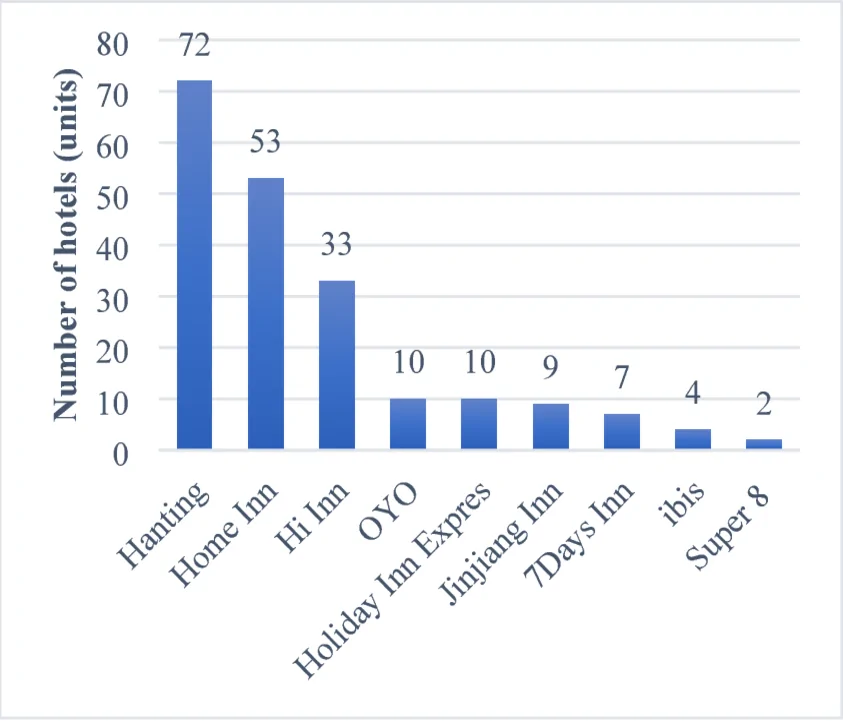
The number of hotel brands selected for research in this article.

This study selected nine economical chain hotel—Hanting, Home Inn, Hi Inn, 7 Days Inn, Ibis, Jinjiang Inn, OYO, Super 8, and Holiday Inn Express—as research targets (Fig. 3). Collectively operating over 5,000 outlets nationwide, these brands capture more than 50% of China’s economy chain hotel market share, ensuring robust representativeness. The study investigated and calculated the carbon emissions of 200 hotels across these nine brands in Hangzhou (Fig. 4)., The findings provide valuable insights to guide low-carbon transition strategies for the hotel industry in Hangzhou and serve as a reference for nationwide implementation.

#### Carbon emissions calculation

Based on the literature review on carbon emission accounting, the building life cycle can be categorized into five key stages: production, transportation, on-site installation, renovation, and dismantling. Accordingly, the total carbon emissions of a building can be calculated as follows:

1 $$\:W={W}_{1}+{W}_{2}+{W}_{3}+{W}_{4}+{W}_{5}$$

where W denotes the total carbon emissions of the building; W₁ represents emissions during the production stage; W₂ corresponds to emissions from transportation; W₃ refers to emissions generated in the on-site installation stage; W₄ indicates emissions associated with renovation; and W₅ denotes emissions arising from the dismantling stage. It should be emphasized that the total carbon emissions W defined in this study only covers the non-operational life-cycle stages (production, transportation, construction, renovation, and dismantling). It does not include the operational stage (e.g., energy consumption for heating, cooling, lighting, appliances, etc.).

1. Production stage.
  This stage accounts for the carbon emissions generated during the extraction, processing, and manufacturing of primary building materials. The calculation scope focuses on materials that contribute significantly to embodied carbon and are directly related to building volume and morphology, including: concrete (for the main structure), steel reinforcement, and masonry blocks (for envelope walls). The carbon emissions are calculated as:
  2 $$\:{W}_{1}={\sum\:}_{i=1}^{n}{M}_{i}{F}_{i}$$
  where M_i_ is the consumption of the i-th material (kg or m³), and F_i_ is the corresponding carbon emission factor (kgCO₂/unit quantity of material).
2. Transportation stage.
  This stage accounts for the carbon emissions generated during the transportation of building materials from production sites to the project location. The calculation scenario assumes that all materials are transported by road using light-duty diesel trucks with a rated load capacity of 2 tons, considering full load conditions. The carbon emissions are calculated as:
  3 $$\:{W}_{2}={\sum\:}_{i=1}^{n}{M}_{i}{D}_{i}{T}_{i}$$
  where D_i_ represents the average transportation distance of the i-th material (km), and T_i_ is the carbon emission factor per unit weight of the i-th material for its transportation mode (kgCO₂/kg·km).
3. Construction stage.
  This stage accounts for the carbon emissions generated by mechanical energy consumption and human activities at the construction site. The calculation scope includes the energy consumption of mechanical equipment used in major civil and installation processes, as well as the basal metabolic emissions of construction personnel.The carbon emissions are calculated as:
  4 $$\:{W}_{3}={C}_{j}+{C}_{r}$$
  5 $$\:{C}_{j}={E}_{j}{R}_{j}{F}_{j}$$
  6 $$\:{C}_{r}={Q}_{r}{F}_{r}$$
  where C_j_ is the carbon emissions from construction machinery; C_r_ is emissions from labor; E_j_ is the quantity of the j-th construction machine; R_j_ is its energy consumption per unit shift; F_j_ is the emission factor of the corresponding energy (kgCO₂/kWh); Q_r_ is the labor consumption (man-days); and F_r_ is the emission factor per labor unit (kgCO₂/man-day).
4. Renovation stage.
  [52]This stage is specifically established to address the frequent “secondary decoration” characteristic of economy chain hotels, accounting for the incremental carbon emissions generated by brand upgrades or room renovations. The calculation scenario encompasses the production, transportation, and construction of decorative materials^87^. The carbon emissions are calculated as:
  7 $$\:{W}_{4}={C}_{41}+{C}_{42}+{C}_{43}$$
  8 $$\:{C}_{41}={\sum\:}_{c=1}^{n}{M}_{c}{F}_{c}$$
  9 $$\:{C}_{42}={\sum\:}_{c=1}^{n}{M}_{c}{D}_{c}{T}_{c}$$
  10 $$\:{C}_{43}={\sum\:}_{x=1}^{m}{c}_{x}$$
  11 $$\:{C}_{x}={\sum\:}_{y=1}^{n}{c}_{xy}{Q}_{xy}$$
  where C₄₁ is emissions from decoration material production, C₄₂ from decoration material transportation, and C₄₃ from decoration construction. Mc is the consumption of decoration material c (kg); Fc is its emission factor (kgCO₂/unit); Dc is its average transportation distance (km); Tc is its transport emission factor (kgCO₂/kg·km). Cx denotes emissions from the x-th activity in decoration construction, with c_xγ_ representing the work volume of the y-th quantity, and Q_xγ_ the unit emissions per quantity (y = 1, 2, 3…n)^87^.
5. Dismantling stage.
  The dismantling phase includes building demolition, material transportation, and recycling. Following Li [53], demolition emissions are estimated to be 90% of those in the construction stage, and transportation of demolition items is regarded as the reverse of material transport, also accounting for 90% of transportation emissions. Thus, the calculation is:
  12 $$W_{5} = W_{2} \times 90\% + W_{3} ~ \times 90\%$$

#### Data acquisition for inventory analysis

Due to limitations in data availability, part of the material consumption in this study was estimated. For the production stage, the quantities of concrete and blocks were derived from building volume: block consumption was estimated based on the total wall volume (internal wall volume + external wall volume), while concrete consumption was approximated using the floor slab volume, the material consumption of steel is sourced from research literature, and this study adopts a value of 60 kg/m². During the transportation stage, the carbon emission factor was determined by both the transportation mode and energy type. This study assumed material delivery using light-duty diesel trucks with a load capacity of 2 tons, operating under full-load conditions. Transportation distances were set at 40 km for concrete blocks, 20 km for concrete, and 100 km for other materials. Compared to traditional construction methods, prefabricated construction methods can achieve a carbon emission reduction ranging from 35% to 15%. Therefore, this study adopts 70% of the carbon emissions of traditional construction methods for calculation^88–90^. For the renovation stage, the consumption of decorative materials, as well as the labor demand (man-days) in the construction stage, were also estimated. The categories of decorative materials and their corresponding average transportation distances were obtained from published literature^87^.

The carbon emission factors are derived from previous literature and the carbon emission calculation standards officially published by China. The result is shown in Table 3.

**Table 3:** Data sources of carbon emission factors.

Caculation Index	Carbon Emission Factor	Date Sources
Concrete blocks factors	212 kgCO₂/m³	^91^
Concrete factor	295 kgCO₂/m³	
Transportation factors	0.286 kgCO₂/(t·km)	
Steel bars	2208 kgCO₂/t	
doors and windows	194 kgCO₂/m²	
Prefabricated wall panels factor	575.07 kgCO₂/m³	^92^
Prefabricated floor slabs factor	817.72 kgCO₂/m³	
labor carbon emission factor	1.11 kgCO₂/labor-day	^93^
consumption of steel	60 kg/m²	^94^

The assembly rate of prefabricated hotel buildings is assumed to be 70%. For the following reasons: (1) Policy Compliance: A 70% prefabrication rate aligns with the medium-to-high requirements for public and commercial buildings, serving as a policy-representative benchmark. (2) Technical Feasibility: For economy chain hotels, industry practices demonstrate that adopting prefabricated components for main structures (slabs, walls) and partial interior finishes to achieve a 70% prefabrication rate is both technically mature and economically viable. For the determination of the anthropogenic carbon emission factor, this paper does not consider differences in job types and labor intensity for the time being. Instead, the carbon emissions from residential energy consumption are converted into carbon emissions per labor-day. Given that China’s per capita carbon emissions range from 2,203 to 2,333 kg/year, with a mean value of 2,268 kg/year, the carbon emission factor for the construction phase is derived as 1.11 kgCO₂ per person per labor-day.

### Calculation method of carbon emission intensity

For comparative analysis of the carbon emissions per unit, elements such as building area, building height, floor-to-floor height, number of residential units, and usable area can be selected^95^.Based on the above conclusion, this paper chooses to use the carbon emission per unit volume of the building to compare the carbon emission intensity of 200 economy chain hotels. The characteristic of carbon intensity is the carbon emission per unit building area^96–98^. It is a normalized indicator derived by dividing the total carbon emissions by the building volume. CEI enables comparison across buildings of different sizes.Therefore, the calculation formula for the carbon emission intensity per unit volume is as follows:

13 $$\:P=\frac{W}{V}$$

Where * P* is carbon emission intensity per unit volume of the building(kgCO_2_/m³), * V* is the volume of building(m³).

### Form classification

To comprehensively analyze the impact of building shape on carbon efficiency, this paper categorizes the 200 sample hotels based on factors such as shape coefficient (S/V), building height, number of floors, and window-to-wall ratio, resulting in five distinct building shape types: Type A (rectangular), Type B (polygonal), Type C (curvilinear), Type D (courtyard), and Type E (irregular).

Type A – Rectangular: The plan is square and regular, with large standard floor areas, a small shape factor, a moderate window-to-wall ratio, and a simple, regular facade. This type is convenient for construction, saves land, and is the most common among economy chain hotels.

Type B – Polygonal: The plan consists of multiple linear segments, forming “L,” “Z,” or “V” shapes, typically resulting from site boundary constraints or street frontage requirements. The shape factor is moderate, floor areas vary significantly across levels, and the window ratio shows slight variations. While this form enhances architectural identity and interior spatial diversity, it also leads to larger external wall areas and more complex structural issues.

Type C – Curvilinear: At least one major facade or the entire building footprint is curved or arc-shaped (e.g., sector, semi-circle, or partial curved surface). The shape factor is large, the standard floor height varies with the curvature, and windows must be custom-made according to the curve, resulting in a wide range of window-to-wall ratios.

Type D – Courtyard: The plan is arranged in a “□” (enclosed) layout, with spaces organized around one or more inner courtyards or atriums, commonly found in centralized hotel buildings. The shape factor increases due to the courtyard openings; the standard floor area is large, but the usable floor area is affected by the atrium; windows face both outward and inward. This form facilitates natural lighting and ventilation but increases the envelope area and material waste during the renovation stage.

Type E –Irregular: The plan is irregular, lacking a clear geometric order, often formed by interlocking volumes, split levels, cantilevers, etc. The facade features frequent concave-convex changes, and the window-to-wall ratio fluctuates significantly.

## Results

### Carbon emissions calculation results

The analysis in this section is based on the following clearly defined variables: Carbon Emission Intensity (CEI, unit: kgCO₂/m³), which serves as the core comparative metric; the Building Shape Factor (S/V), defined as the ratio of the building’s external surface area to its volume, representing the key three-dimensional morphological variable; Carbon Reduction Quantity (ΔC = CC-PC) and Carbon Reduction Efficiency (ΔC/CC).

In this study, a comprehensive approach was adopted to assess the lifecycle carbon emissions of buildings, encompassing material production, transportation, construction, interior finishing, and demolition. The carbon emissions for each stage of the hotel buildings are detailed in the appendix. Among both prefabricated and traditional constructions, the Holiday Inn Express Hangzhou Binjiang Olympic Sports exhibited the highest carbon emissions at 6,181,296.539 kgCO₂ and 6,304,716.531 kgCO₂, respectively, with Type A. The lowest emissions in prefabricated hotels were observed at the OYO Hangzhou Jinyao Hotel (116,359.7055 kgCO₂, rectangular layout), while the traditional construction with the lowest emissions was the Hi inn Hotel (Hangzhou Yuhuangshan Baguatian) at 124,686.8395 kgCO₂, which is also classified as Type A. The estimated lifecycle carbon emissions show a general proportionality to floor area, indicating a potential linear relationship.

### Calculation results of carbon emission intensity

**Fig. 5:**
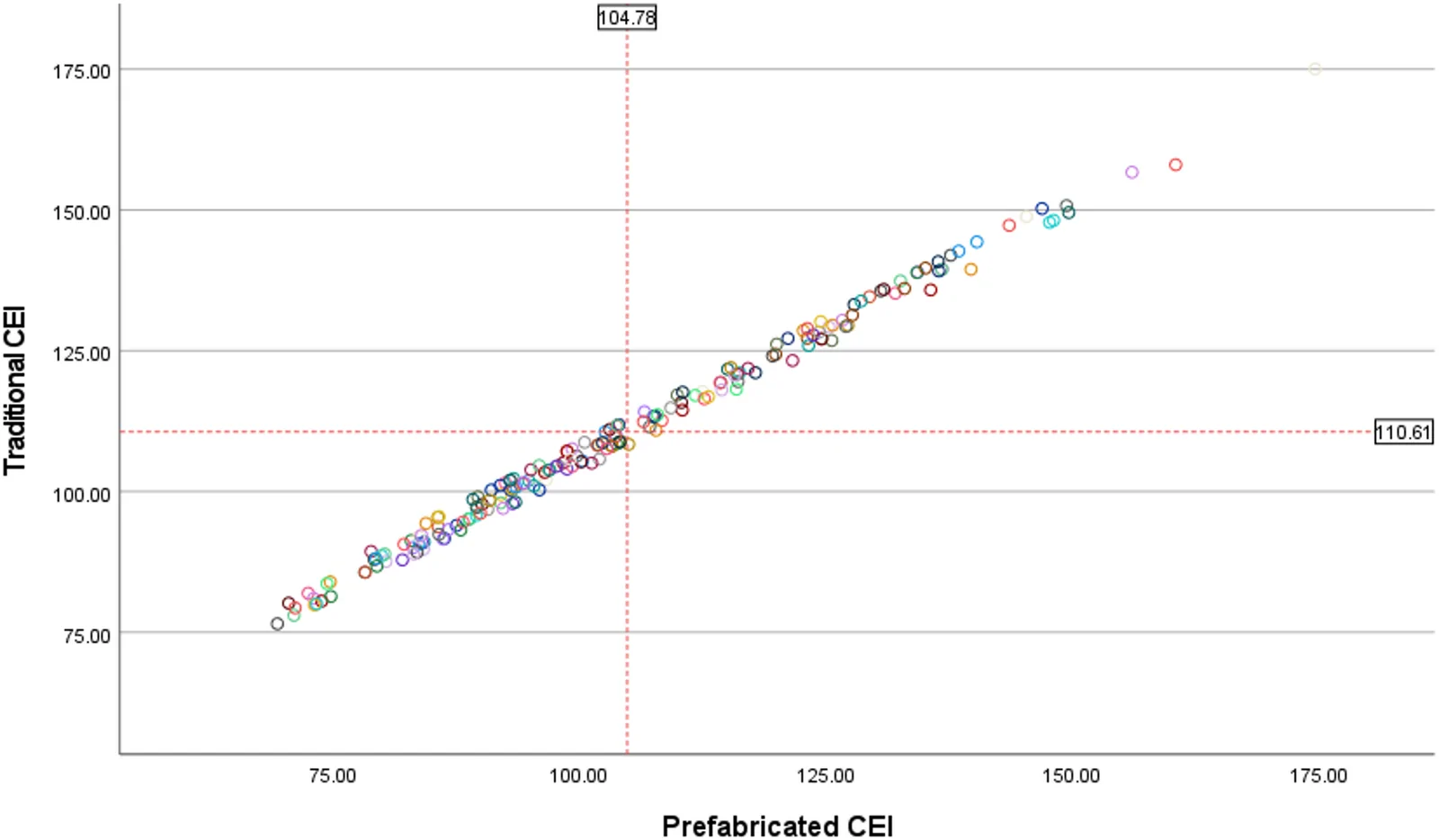
The morphological induction of CEI.

**Fig. 6:**
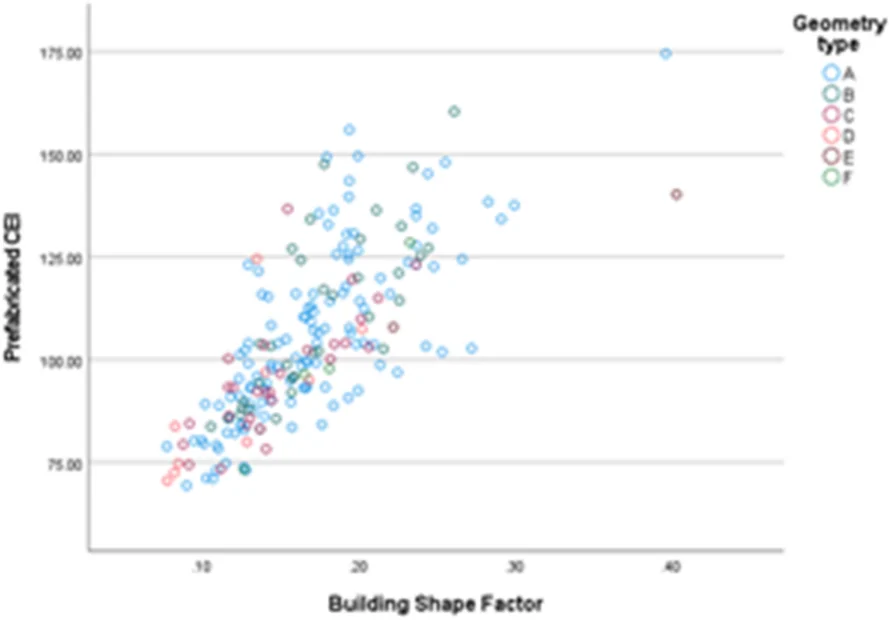
The relationship between building shape factor and prefabricated CEI.

**Fig. 7:**
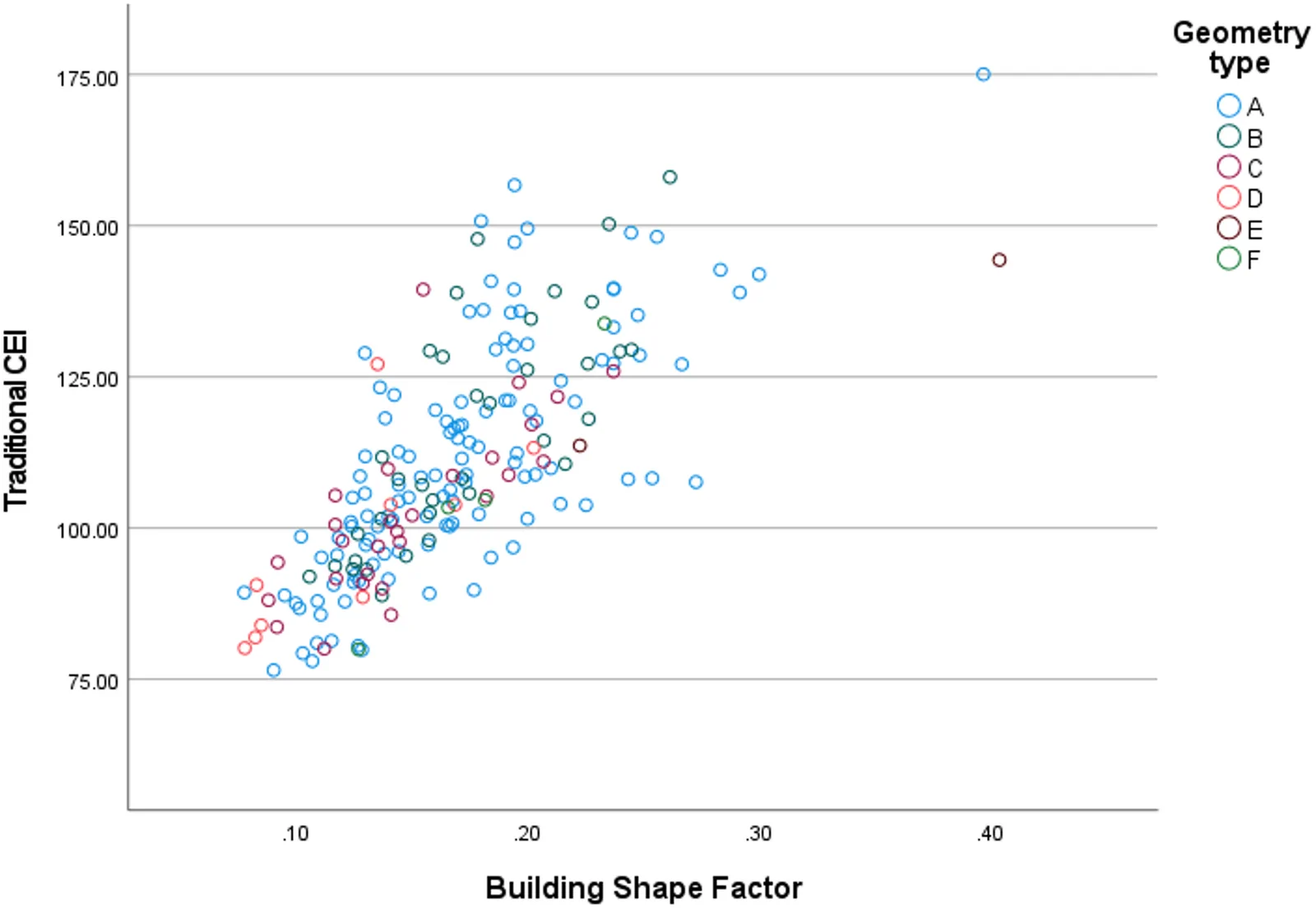
The relationship between building shape factor and traditional CEI.

**Fig. 8:**
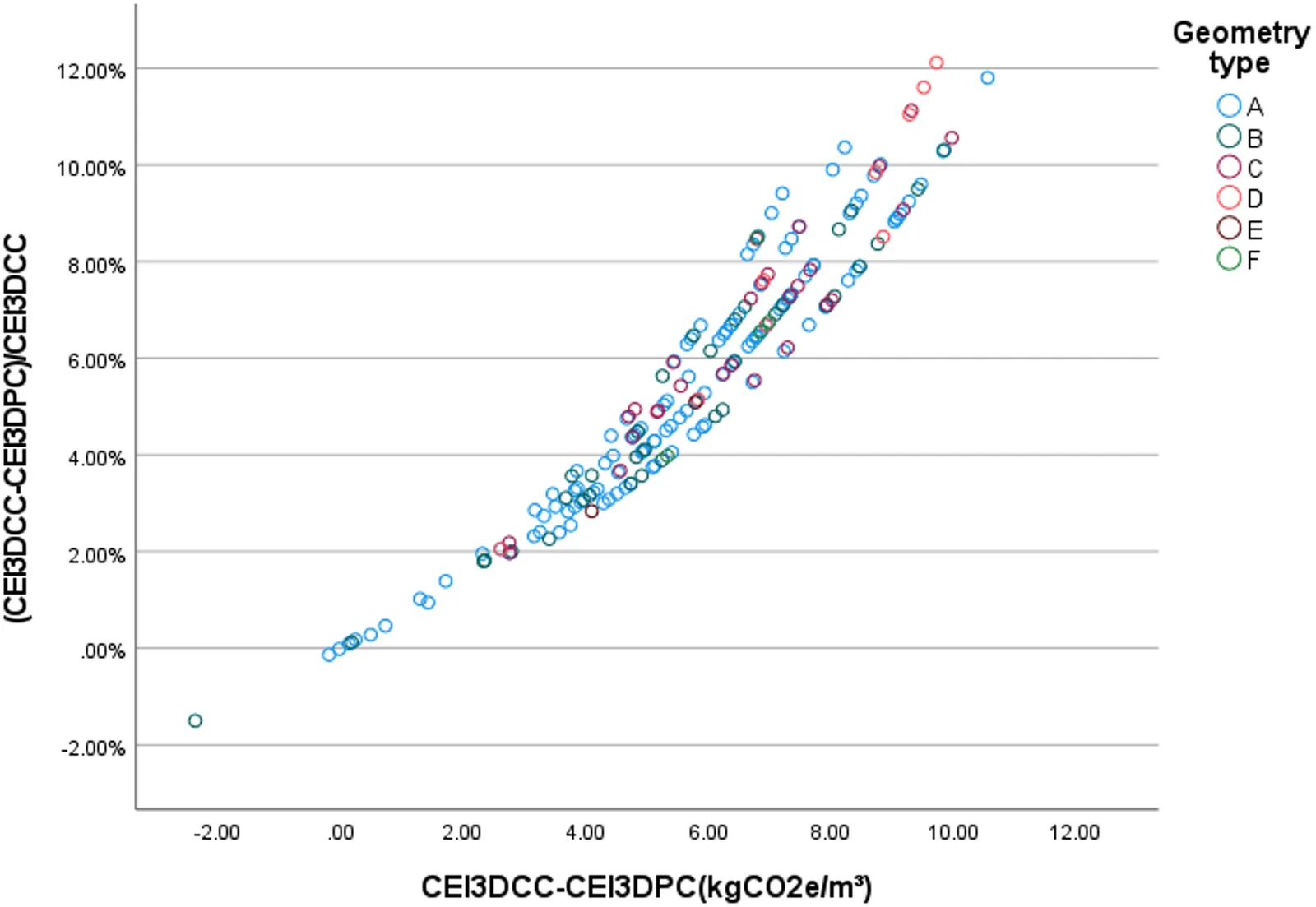
The relationship between (CC-PC): CC and CC-PC.

By dividing the total carbon emissions by the building volume, the study calculated the CEI (Figs. 5, 6 and 7). Among prefabricated buildings, the mean CEI for prefabricated hotels is 108.2 kgCO₂/m³ (standard deviation = 15.3, range: 116.4-174.6), while the mean CEI for traditional hotels is 114.7 kgCO₂/m³ (standard deviation = 16.1, range: 124.7-175.1). The distributions of both datasets exhibit a slight positive skewness. The carbon reduction quantity of buildings is obtained by subtracting prefabricated CEI (CC-PC) from conventional CEI, while the carbon reduction efficiency is calculated by dividing this result by conventional CEI ((CC-PC): CC), The specific relationship is illustrated in the accompanying Fig. 8.

To assess robustness, potential outliers were identified using the 1.5×IQR rule. A small number of outliers (< 5% of the sample) corresponded to hotels with highly complex forms or compact volumes. Their removal did not materially alter the mean CEI, the significance of the CEI-shape factor correlation, or the comparative conclusion on prefabricated construction’s carbon efficiency, confirming the robustness of the core findings.

After empirical analysis of 200 chain hotels, the following two conclusions can be drawn:

1. The study’s analysis of CEI across 200 hotel buildings revealed significant discrepancies in carbon emission between prefabricated and traditional hotels. This inconsistency demonstrates a selective adaptation pattern where architectural spatial forms interact differently with construction technologies: prefabricated systems show superior efficiency when applied to handling curvilinear or irregular layouts, while traditional cast-in-situ techniques prove more carbon-efficient for modular, regular geometries (e.g., rectangular or polygonal forms).The findings indicate that effective low-carbon building strategies must achieve synergistic optimization between spatial morphology and construction technology, rather than promoting any single approach universally.
2. Analysis reveals a consistent positive correlation between building shape factors and CEI for both prefabricated and traditional hotels—the larger the form coefficient, the higher the CEI. For prefabricated hotels, when the form coefficient increases from 0.1 to 0.2, the average CEI rises by 23.7% (from 74 kgCO₂/m³ to 103.5 kgCO₂/m³). Notably, The shape factor (S/V) demonstrates a significant positive correlation with CEI in both groups (prefabricated: *r* = 0.72, *p* < 0.001; traditional: *r* = 0.75, *p* < 0.001). Linear regression indicates that the slope of CEI increase with S/V for prefabricated buildings (β = 0.62) is significantly lower than that for traditional buildings (β = 0.68), with the statistical test for the difference between the two slopes yielding *p* < 0.05. Assuming that the hotel traditional construction method is replaced with a prefabricated construction method, the carbon emission intensity per unit volume will be lower. Therefore, prefabricated buildings are more suitable for complex plans form in new construction (renovation).

### Comparison of CEI between different brands and district

This study conducted a systematic comparison of Carbon Emission Intensity (CEI) across 200 economy chain hotels in Hangzhou (covering 9 brands: Hanting, Home Inn, Hi Inn, 7 Days Inn, Ibis, Jinjiang Inn, OYO, Super 8, and Holiday Inn Express). Through lifecycle carbon emission modeling combined with building shape factors (S/V) analysis, the study reveals the driving mechanisms behind CEI variations among brands (Table 4).

**Table 4:** Carbon emission information of each brand.

Brand	Average traditional CEI(kgCO_2_/m^3^)	Average Prefabricated CEI(kgCO_2_/m^3^)	Average (CC-PC)/ CC
Hanting	114.37	108.95	5.03%
Home Inn	107.71	101.54	6.12%
Hi inn	102.06	95.15	7.13%
OYO	117.86	111.04	6.40%
Jinjiang Inn	118.20	113.63	4.20%
Holiday Inn Express	106.63	102.42	4.35%
7Days Inn	124.39	119.50	4.16%
Ibis	113.65	108.60	5.06%
Super 8	88.57	81.31	8.23%

Cluster 1 (Low-Carbon Group): Super 8, Hi Inn, Holiday Inn Express, CEI Range: 88.57-106.63 kgCO₂/m³, these brands demonstrate superior carbon efficiency, primarily attributable to their adoption of compact rectangular floor plans and high prefabrication rates (≥ 65% assembly rate). Cluster 2 (Medium-Carbon Group): Hanting, Home Inn, Ibis, CEI Range: 107.71-113.65 kgCO₂/m³. Mid-range carbon intensity reflecting balanced design approaches. Cluster 3 (High-Carbon Group): 7 Days Inn, Jinjiang Inn, OYO, CEI Range: 117.86-124.39 kgCO₂/m³. Elevated emissions correlate with complex architectural geometries (e.g., curvilinear forms, irregular layouts) that increase material usage and construction complexity. Hi Inn and Super 8 showed the lowest CEI values among the nine brands. A possible explanation is their highly standardized modular guest room units and compact floor plans, but this remains a hypothesis that requires further investigation with controlled variables. 7 Days Inn and Jinjiang Inn exhibited the highest CEI values. One potential contributing factor is their relatively complex building forms, but other unmeasured confounders (e.g., older construction periods, different technical standards, or district‑level policy variations) cannot be ruled out.

**Fig. 9:**
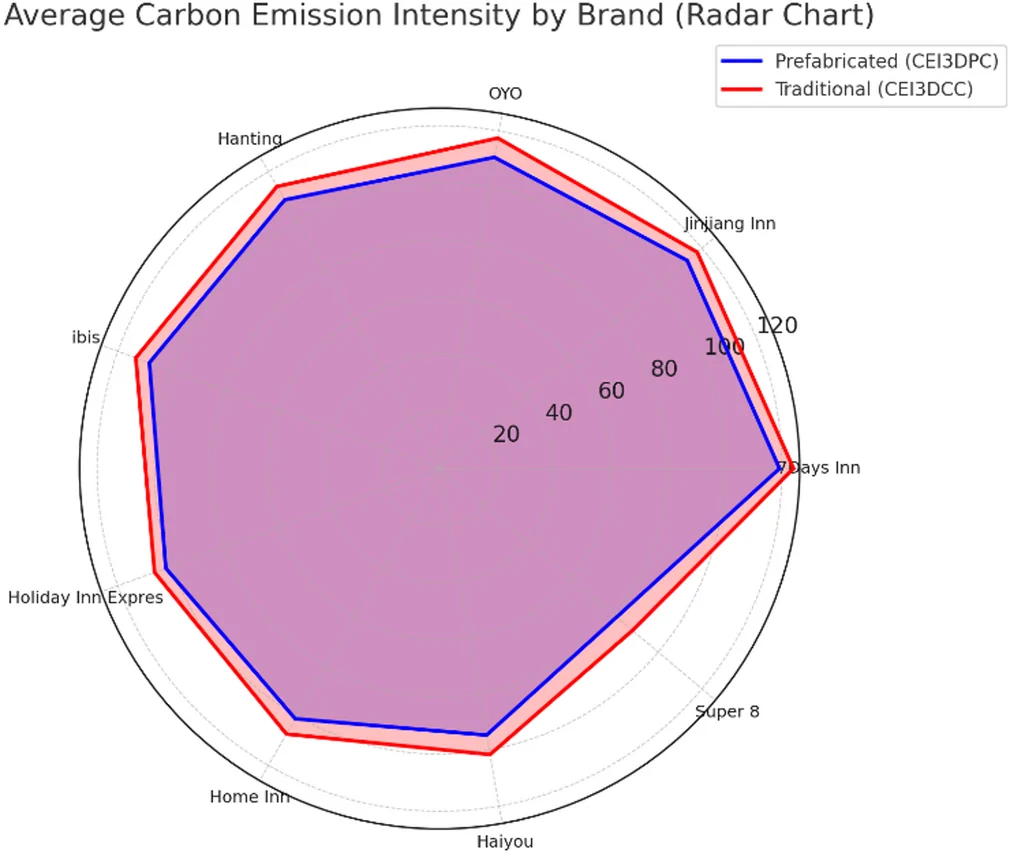
Average Carbon Emission Intensity by Brand (Radar Chart).

**Fig. 10:**
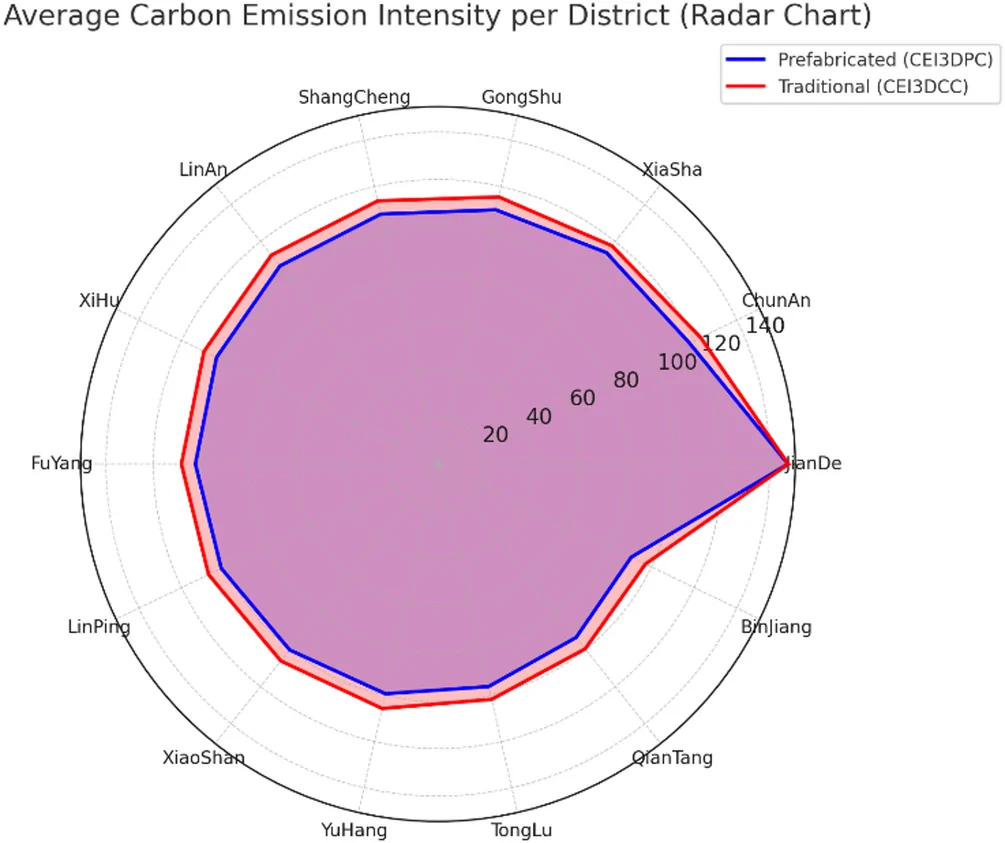
Average carbon emission intensity per district (radar chart).

An inter-brand analysis of carbon emission intensity (CEI) among economy chain hotels in Hangzhou revealed significant variability in both construction methods and design preferences (Fig. 9). Brands such as Hanting exhibited the highest average CEI values, with CEI3DPC and CEI3DCC reaching 134.2 kgCO₂e/m³ and 139.6 kgCO₂e/m³ respectively, while others like Yitel and Hi Inn maintained much lower emissions, with prefabricated CEI values below 100 kgCO₂e/m³. In approximately 85% of the brands analyzed, traditional construction (CEI3DCC) resulted in higher emissions compared to prefabricated structures (CEI3DPC), with an average increase ranging from 3% to 7%. These trends suggest that prefabricated construction methods offer a consistent advantage in reducing carbon intensity. Additionally, brands with lower CEI values often favored more compact and regular geometric configurations—such as polygonal floor plans and lower building shape factors—highlighting the influence of architectural design on environmental performance. Collectively, these findings underscore the impact of brand-level construction strategies on carbon outcomes and support the promotion of prefabricated technologies for sustainable hotel development.

A comparative analysis of 3D morphology-related CEI was conducted across economy chain hotels in various districts of Hangzhou (Fig. 10). Two metrics were evaluated: CEI3DPC (kgCO₂e/m³) for prefabricated buildings and CEI3DCC (kgCO₂e/m³) for traditional constructions. The results revealed notable spatial differences, with the highest average CEI3DPC observed in Shangcheng District (139.8 kgCO₂e/m³), followed by Xiacheng and Binjiang, while Yuhang and Xihu exhibited the lowest values. Across all districts, CEI3DCC values were consistently higher than CEI3DPC in approximately 78% of cases, indicating that prefabricated construction methods generally produce lower carbon emissions related to building morphology. For instance, in Gongshu District, CEI3DPC averaged 122.4 kgCO₂e/m³ compared to 126.1 kgCO₂e/m³ for CEI3DCC, reflecting a 3.02% increase. The magnitude of difference between the two CEI measures varied by district, suggesting potential influences from local building geometry, form factors, or planning regulations. These findings underscore the importance of considering spatial and morphological factors in carbon reduction strategies for urban hotel development.

Although building morphology and construction technology have been identified as key correlated variables in this study, other potential influencing factors should not be overlooked: (1) The impact of temporal and technological dimensions. Differences in construction periods may lead hotels to comply with varying technical standards. Earlier projects typically adhere to lower energy efficiency requirements and equipment performance, which constitutes a historical cause of carbon emission disparities. (2) The potential role of regional and systemic factors. Variations exist among administrative districts in terms of green building policies, the sustainability level of local supply chains, and traditional construction practices. These regional “soft environment” factors may indirectly affect carbon intensity. Furthermore, although differences in brand-specific selection criteria for HVAC, lighting, and other equipment systems fall outside the operational phase boundary of this study, their efficiency may interact with building morphology. This represents an important dimension to be integrated into future whole-life cycle assessments.

## Discussion

This study highlights the multifaceted relationship between construction methods, architectural morphology, and carbon emission performance in the economy hotel sector. One of the most significant findings is the non-uniform efficiency of prefabricated versus traditional construction across varying spatial forms. While prefabrication generally demonstrated lower lifecycle carbon emissions, its performance advantage was particularly pronounced in buildings with curvilinear or irregular geometries.

This suggests that prefabricated systems are more adaptable to complex architectural configurations, the carbon efficiency advantage demonstrated by prefabricated construction in complex geometric forms stems from its systematic reconfiguration of the “design-production-construction” workflow, primarily achieved through the following interrelated mechanisms: (1)Material Flow Optimization: BIM-driven precision manufacturing in factory settings drastically reduces material cutting waste generated during on-site adaptation to complex forms in traditional construction, thereby lowering embodied carbon emissions at the source; (2)Construction Flow Simplification: Complexity in morphology is shifted to the design and manufacturing stages, simplifying on-site operations to standardized hoisting and assembly. This significantly reduces the need for intricate formwork, elevated work in complex spaces, and heavy machinery energy consumption typical of traditional methods, thereby cutting carbon emissions during the construction phase; (3)Quality Flow Control: The higher component accuracy and consistency ensured by factory production provide a reliable foundation for achieving designed performance (e.g., airtightness, thermal performance) in complex building envelopes, potentially reducing long-term operational carbon costs associated with construction defects. Thus, the advantage of prefabricated construction lies in transferring the resource consumption required to address morphological complexity from the high-uncertainty construction site to the controlled and efficient digital factory, achieving systemic carbon savings across the entire process.

The reduction factor is used as a computational parameter for prefabricated hotels in hangzhou, not as a premise for our findings. Our core conclusion—that prefabrication’s carbon advantage is morphology-dependent—is derived from comparative analysis across 200 samples, not from the assumed factor itself. Meanwhile, to examine whether the construction method moderates the impact of morphology on carbon intensity, we established a linear regression model incorporating an interaction term (S/V × Construction Method), as shown in Table 5. The analysis revealed that the shape factor (S/V) has a significant positive correlation with CEI (β = 0.68, *p* < 0.001). The key interaction term (S/V × Prefabricated Construction) has a coefficient that is significantly negative (β = 0.62, *p* = 0.922). This indicates that, compared to traditional construction methods, the rate of increase in CEI with S/V under prefabricated construction is significantly reduced by 0.922 units. In other words, the regression slope for prefabricated buildings (β = 0.62) is significantly lower than that for traditional buildings (β = 0.68), and this difference is supported by statistical testing (*p* < 0.05).

This implies that prefabricated construction is less sensitive to geometric complexity, offering greater resilience in carbon performance when faced with intricate design requirements. At the brand level, the variation in CEI among hotel operators reflects organizational differences in design standardization and construction adoption. Low-carbon clusters (e.g., Super 8, Hi Inn) consistently leveraged compact, modular designs and higher prefabrication rates, achieving up to 8.23% emission reductions. In contrast, high-carbon brands (e.g., 7 Days Inn, Jinjiang Inn) favored more architecturally ambitious forms, which—while potentially improving aesthetic or experiential quality—resulted in greater material usage and carbon intensity.

**Table 5:** Testing for interaction effects in regression models.

Model	Unstandardized Coefficients B	Standardized Coefficients Beta	t	Sig.(*p*)
(Constant)	97.18	–	19.346	< 0.001
Shape Factor	48.288	0.192	2.782	0.006
Group(P and C)	− 5.162	− 0.129	− 0.727	0.468
Shape Factor × Group	− 2.396	− 0.018	− 0.098	0.922

^a^Dependent Variable: Traditional Carbon Emission Intensity.

District-level differences in CEI (Fig. 10) point to urban spatial factors such as site constraints, planning regulations, and local construction practices that further modulate carbon outcomes. The consistent advantage of prefabrication in 78% of the surveyed districts underlines the general applicability of this method for urban sustainability, especially in areas with complex morphological conditions. Overall, the discussion confirms that effective carbon reduction strategies cannot rely solely on promoting a single construction method, but must instead align building technology with design geometry and urban context to optimize lifecycle performance.

The literature-derived reduction factor is used as a computational parameter for prefabricated hotels, not as a premise for our findings. Our core conclusion—that prefabrication’s carbon advantage is morphology-dependent—is derived from comparative analysis across 200 samples, not from the assumed factor itself. A methodological limitation of this study concerns the simplified material inventory adopted for the production-stage carbon calculation. By assuming that all 200 hotels share identical construction systems and that only the quantities of concrete, steel, and blocks vary with building volume, the analysis does not capture more nuanced sources of embodied carbon. These include: (1) differences in material efficiency between prefabricated and traditional construction, (2) alternative material specifications that prefabrication enables, (3) embodied carbon from secondary structural elements, and (4) variability in connection details and joint materials between prefabricated components. Consequently, the reported carbon reduction advantages of prefabricated hotels should be interpreted as conservative estimates that primarily reflect differences in construction-process emissions (transport, on-site installation, renovation) rather than a full accounting of embodied carbon benefits from material optimization. Future research should incorporate component‑level bill of quantities data from actual projects or BIM models to refine the comparison.

This study assumes a uniform prefabrication assembly rate of 70% for all prefabricated hotels, based on policy benchmarks and technical feasibility. In reality, assembly rates vary across projects due to differences in design, cost, and construction capabilities. A fixed rate may not capture the full range of carbon performance. Future research should conduct sensitivity analyses using different assembly rates (e.g., 30%, 50%, 70%, 90%) to test the robustness of morphology-dependent findings. However, we note that the main conclusion—prefabrication offers greater carbon efficiency for complex forms—is likely to hold as long as the assembly rate for prefabricated hotels is consistently higher than that for traditional ones, which is a reasonable baseline assumption.

## Conclusion

This study provides a preliminary, estimation-based lifecycle assessment (excluding the operational phase) of carbon emissions across 200 economy chain hotel buildings in Hangzhou, with a focus on the comparative performance of prefabricated and traditional construction methods in relation to architectural morphology and brand-level design choices. The findings suggest that prefabricated construction methods consistently may outperform traditional ones in terms of carbon emission intensity (CEI), particularly when applied to buildings with complex spatial forms such as curvilinear or irregular layouts. This suggests that prefabrication is not only viable but also potentially preferable in scenarios where architectural complexity would otherwise lead to significant material waste or on-site inefficiencies. Importantly, the analysis also suggests that traditional cast-in-place construction remains more carbon-efficient for simple, modular forms, such as rectangular or polygonal layouts, indicating that neither method is universally superior but rather context-dependent.

A key insight from the study is the observed positive correlation between building shape factor and CEI, implying that more geometrically complex forms result in higher emissions due to increased surface area and thermal exchange. Notably, prefabricated buildings demonstrated a lower sensitivity to rising shape factors, indicating a more stable performance under morphologically challenging conditions. The inter-brand comparison further revealed that low-carbon performers, such as Super 8 and Hi Inn, consistently applied compact geometries and high prefabrication rates, leading to substantial emission reductions—up to an estimated 8.23% compared to their traditional counterparts. Conversely, brands with more complex and expressive architectural designs (e.g., Jinjiang Inn, 7 Days Inn) exhibited significantly higher CEI values. These differences underscore the influence of corporate design strategies on environmental performance.

Finally, spatial analysis across districts showed that urban planning context and local building practices also affect CEI outcomes, reinforcing the need for location-specific carbon mitigation strategies. In conclusion, this study highlights the importance of a synergistic approach to low-carbon hotel development that integrates construction technology, spatial form, and brand-level standardization. Rather than promoting prefabrication as a one-size-fits-all solution, the results advocate for context-aware design decisions that align construction methods with architectural and urban conditions to maximize lifecycle carbon efficiency.Significantly, the conclusions of this study are derived from economy chain hotels in Hangzhou, China, and their applicability is constrained by contextual boundaries: (1) geographical/climatic factors (regional climate, supply chains, construction practices), (2) policy/regulatory frameworks (China’s carbon and prefabrication policies), and (3) building typology (standardized economy chain hotels versus luxury or complex buildings). Therefore, direct extrapolation of prefabrication advantages or form-technology relationships to other regions, policies, or building types requires caution. This study provides an empirical and analytical framework for low-carbon hotel design in similar contexts, with broader generalizability requiring future research across diverse cities, typologies, and dynamic regional data.

Furthermore, the conclusions of this study are primarily derived from a sample of a specific hotel type (economy chain hotels) in Hangzhou. The results are based on data estimations, do not account for the influence of other factors, and the unified emission factors fail to reflect regional grid differences, local material supply chains, and the impact of technological advancements over time. Therefore, directly extrapolating the advantages of prefabricated construction to other contexts should be approached with caution. Future research could expand to include more cities and hotel types, employ more precise measured data and dynamic emission factors, and control for additional design variables to develop a more universally applicable “form-technology-carbon efficiency” decision-making model. The material inventory for the production stage is limited to concrete (for columns, beams, slabs), steel reinforcement, and masonry blocks (for infill walls)—materials directly correlated with building volume and common to both construction systems. A key simplification is made: all hotels are assumed to use identical material types and construction methods (reinforced concrete frame with masonry infill) for these components. Therefore, the estimated production emissions (W₁) are driven primarily by component quantities (scaling with building dimensions), rather than by differences in material efficiency, component design, or construction techniques between prefabricated and conventional methods. This assumption ensures a consistent baseline for morphological comparison across 200 samples, but inevitably underestimates the embodied carbon benefits that prefabrication may offer (e.g., reduced material waste, optimized designs, or lightweight precast panels). Future research should move beyond the simplified material inventory adopted here (which assumed identical construction systems across all hotels) and incorporate component‑level data—such as actual reinforcement ratios, precast concrete mix designs, and connection details—to better quantify the embodied carbon benefits of prefabrication beyond volume‑driven differences.

## Data Availability

The data that support the findings of this study are available from the corresponding author upon reasonable request.
